# ROLE of IGF-1 System in the Modulation of Longevity: Controversies and New Insights From a Centenarians' Perspective

**DOI:** 10.3389/fendo.2019.00027

**Published:** 2019-02-01

**Authors:** Giovanni Vitale, Giuseppe Pellegrino, Maria Vollery, Leo J. Hofland

**Affiliations:** ^1^Laboratorio Sperimentale di Ricerche di Neuroendocrinologia Geriatrica ed Oncologica, Istituto Auxologico Italiano IRCCS, Milan, Italy; ^2^Department of Clinical Sciences and Community Health, University of Milan, Milan, Italy; ^3^Faculty of Medicine, University of Campania “Luigi Vanvitelli”, Naples, Italy; ^4^ASP Redaelli Golgi, Milan, Italy; ^5^Division Endocrinology, Department of Internal Medicine, Erasmus Medical Center, Rotterdam, Netherlands

**Keywords:** IGF-1, insulin, longevity, centenarians, caloric restriction, aging, rehabilitation medicine

## Abstract

Human aging is currently defined as a physiological decline of biological functions in the body with a continual adaptation to internal and external damaging. The endocrine system plays a major role in orchestrating cellular interactions, metabolism, growth, and aging. Several *in vivo* studies from worms to mice showed that downregulated activity of the GH/IGF-1/insulin pathway could be beneficial for the extension of human life span, whereas results are contradictory in humans. In the present review, we discuss the potential role of the IGF-1 system in modulation of longevity, hypothesizing that the endocrine and metabolic adaptation observed in centenarians and in mammals during caloric restriction may be a physiological strategy for extending lifespan through a slower cell growing/metabolism, a better physiologic reserve capacity, a shift of cellular metabolism from cell proliferation to repair activities and a decrease in accumulation of senescent cells. Therefore, understanding of the link between IGF-1/insulin system and longevity may have future clinical applications in promoting healthy aging and in Rehabilitation Medicine.

## Introduction

Aging is defined as a physiological decline of biological functions in the body with a progressive decline or loss of adaptation to internal and external damaging. In humans the aging phenotype is extremely heterogeneous and can be described as a complex mosaic resulting from the interaction of several stochastic and environmental events, genetic, and epigenetic alterations accumulated throughout the lifetime. Despite its enormous complexity, the molecular basis of aging is limited to few highly evolutionarily conserved biological mechanisms responsible for body maintenance and repair (1).

During the last 3 decades one of the most discussed topics in gerontology is the role of the growth hormone (GH)/insulin-like growth factor-1 (IGF-1)/insulin system in the regulation of longevity. Accumulating evidence suggests that this pathway plays an essential role in the pathogenesis of several age-related diseases including cancer, dementia, cardiovascular, and metabolic diseases (2–4).

In animal models it was shown that down-regulation of the GH/IGF-1/insulin system significantly prolongs the lifespan. However, in humans data are contradictory (5, 6).

This review describes the latest advances in the research of the IGF-1 system and modulation of longevity, hypothesizing that the endocrine and metabolic adaptation observed in centenarians and in mammals during caloric restriction may be a physiological strategy for extending lifespan through a slower cell growing/metabolism, a better control in signal transmission and physiologic reserve capacity and a decrease in accumulation of senescent cells. A review of the literature was conducted using PubMed database with the following keywords: “IGF-1” or “IGF-I” and “longevity.” The search included articles published in the English language between January 2008 and August 2018.

## IGF-1 System and Longevity in Animal Models

**Figure 1:**
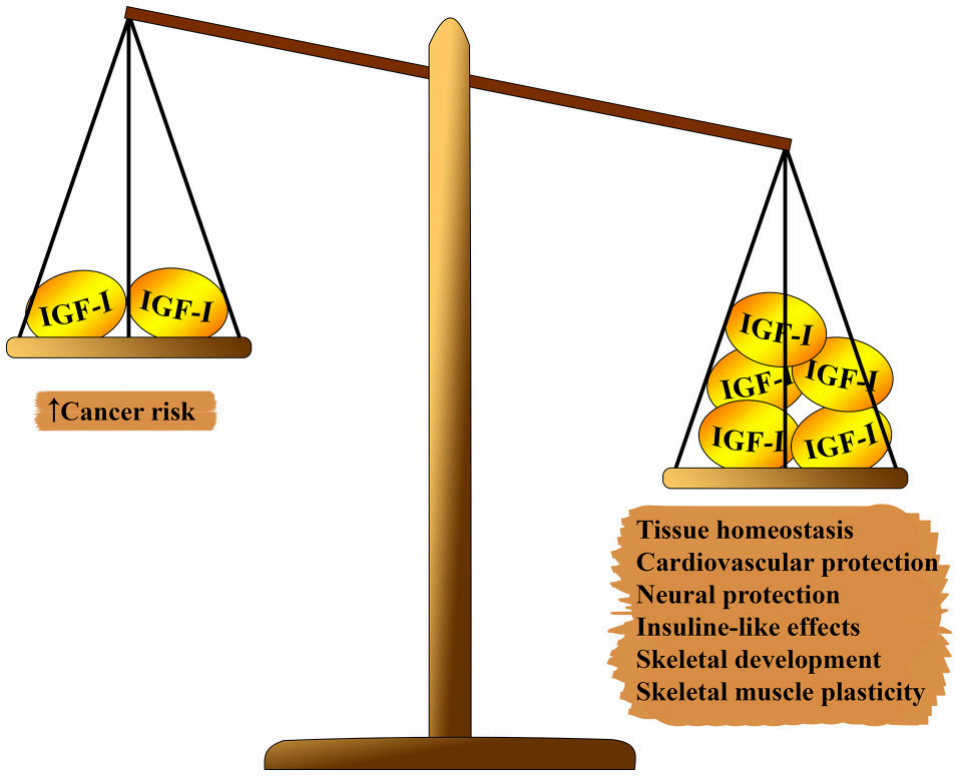
Pleiotropic effects of IGF-1 on health status.

**Figure 2:**
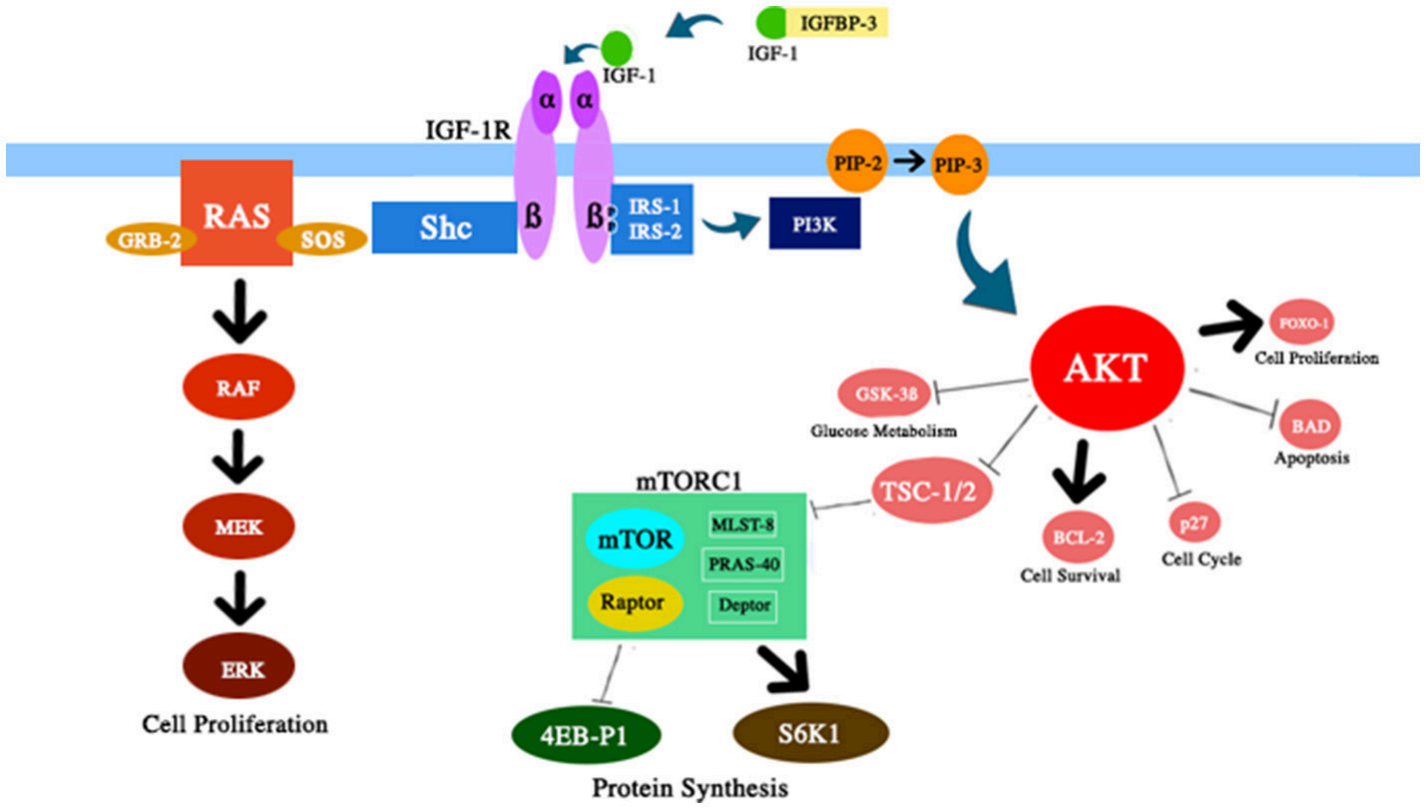
Schematic and simplified representation of the several components of the IGF-1/PI3K/AKT/mTOR pathway discussed in this review. IGF-1 increases the activity of AKT protein with relevant effects on cell survival and proliferation, glucose metabolism and protein synthesis.

IGF-1 system has several pleiotropic effects on biological aging (Figure 1). IGF-1 plays a relevant role in fetal development, growth during childhood and adolescence, and adult tissue homeostasis. In addition, IGF-1 seems to have atheroprotective actions, neural protective, and insulin-like effects (at high concentrations) and to regulate skeletal metabolism and muscle regeneration. Nevertheless, IGF-1 is a main risk factor in several tumors due to its potent proliferative activity, mainly through the modulation of cell cycle, apoptosis, and cell survival (7–9). Most of these effects are mediated through the interaction with insulin receptor substrate (IRS)-1 and-2 and the modulation of the PI3K/AKT/ mammalian target of rapamycin (mTOR) pathway (Figure 2).

Several preclinical studies reported that mutation in genes controlling the GH/IGF-1/insulin signaling pathway can significantly increase lifespan in both invertebrate and vertebrate animal models (5, 6).

### Invertebrate Models

In invertebrates, the insulin/IGF-like cascade is regulated by several peptides, able to interact with a single, common insulin/IGF-1-like receptor.

In the nematode Caenorhabditis elegans the insulin/IGF-like pathway consists of several proteins encoded by the genes daf-2 (insulin/IGF-1 receptor-like protein), age-1 (encoding the catalytic subunit of PI3K), akt-1, akt-2, pdk-1, sgk-1 (serine-threonine kinases), daf-16 (forkhead transcription factor and the major target of insulin-like signaling in Caenorhabditis elegans), skn-1 (oxidative-stress-responsive transcription factor) and daf-18 (PTEN, a phosphatase, involved in inhibition of the AKT signaling pathway). The reduced activity of daf-2, age-1, akt-1, akt-2, pdk-1, sgk-1 genes were shown to downregulate this pathway, and the animals with these mutations were reported to age more slowly and to have an increased lifespan up to 300%. In contrast, the stimulation of the insulin/IGF-like pathway decreases the lifespan of nematodes (10, 11).

In the fruit fly Drosophila melanogaster the insulin/IGF-like signaling consists of the dINR (Insulin /IGF-1 receptor-like protein), the insulin receptor substrate CHICO, the PI3K Dp110/p60, and the PI3K target PKB (akt-1). The flies with mutation in these genes were reported to have significantly increased longevity (12, 13).

Surprisingly, the same molecular mechanisms in different tissues do not influence aging equally. Several studies in nematodes and fruit flies have suggested that reduced insulin/IGF-like signaling in nervous and adipose tissues has the major role in regulation of longevity (14, 15). Although in invertebrate models it was shown that this cascade is relevant in the modulation of lifespan, the influence of insulin/IGF-like signaling on longevity is much more complex in vertebrates, since they have functionally specific insulin and IGF molecules, IGF binding proteins (IGFBPs), IGFBP proteases, GH, multiple receptors and several mechanisms of intracellular signaling with different tissue specific expression (16).

### Vertebrate Models

Several GH/IGF-1 mutant mice have been developed with different targets. The most relevant models are described below.

#### Snell and Ames Mice

Snell and Ames mice are two mouse strains with mutations in the PIT-1 and PROP-1 genes, respectively (17, 18). Since both PIT-1 and PROP-1 proteins are required for the differentiation of pituitary cells that produce GH, prolactin and TSH, both types of homozygous mutant mice lack all three hormones (18). These models have shown remarkable extension of longevity (42–70% more than wild type mice), enhanced insulin sensitivity and lower tumor incidence (19, 20). When Ames dwarfs were exposed to caloric restriction, their lifespan increased even further (21). Although these animals lack three hormones, it has been demonstrated that lifespan extension is mainly influenced by the GH deficiency (22).

#### Lit/lit Mice

Lit/lit mice are GH-deficient, carrying a mutation in the gene which encodes the GH-releasing hormone receptor (GHRHR). These animals were dwarfs, showed increased adiposity, lower tumor incidence and a lifespan increased by 23–25% (19).

#### GH-Releasing Hormone-Knockout (GHRH-KO) Mice

GH-releasing hormone-knockout (GHRH-KO) mice live 43% (in females) and 51% (in males) longer than wild-type animals and share many phenotypic characteristics with Ames dwarf mice, such as enhanced insulin sensitivity, reduction in plasma triglyceride and cholesterol levels, increase in adiposity, plasma leptin, and adiponectin levels (23).

#### The GH-Receptor-Knockout (GHR-KO) Mice

The GH-receptor-knockout (GHR-KO) mice has elevated serum GH levels and very low IGF-1 levels. Also this strain of mice was reported to live 38–55% longer than wild-type (24) and showed attenuation in oxidative stress, as well as a lower and delayed onset of fatal tumors (25). Similar results were observed in df/KO mice, crossing GHR-KO mice and Ames dwarfs, that lacked both GH and GH receptor and maintained extended longevity (26). Unlike wild siblings and Ames dwarf mice, caloric restriction did not further enhance longevity of GHR-KO mice, suggesting that the GH/IGF-1 axis and caloric restriction might have similar or partly overlapping mechanisms for lifespan prolongation (27).

#### GH Receptor Antagonism (GHA)

Not all animal models with suppression of GH/IGF-1 system exhibit an increase in lifespan. The GHA mouse strain is one such example. GHA, generated by the substitution of one amino acid (Gly199 Arg in bovine GH), is able to bind the GH receptor with the same affinity as GH, but does not cause intracellular signaling. The lifespan of GHA mice was not significantly increased (28).

#### IGF-1R^+/−^ Mice

While most of the IGF-1 receptor null mice (IGF-1R^−/−^) die at birth, the animals heterozygous for a mutated allele of the IGF-1 receptor (IGF-1R^+/−^) showed very low serum IGF-1 levels, about 10% smaller size and a 33% increased lifespan in females and 16% in males. However, in this study the wild-type controls lived to only 19 months of age, compromising the interpretation of results (29). More recent studies evaluating the lifespan in another IGF-1R^+/−^ line exhibited a mild 5–10% increase in lifespan, but only in females (30, 31). In addition, the underlying background strain seems to influence the degree of life extension in several murine models (32).

#### A Brain-Specific IGF1-R^+/−^

A brain-specific IGF1-R^+/−^ mutant lived 9% longer than wild-type, underling the relevant role of the neural system in the modulation of longevity (33).

#### Liver-Specific IGF-1-Disrupted Mice (LI-IGF-1^−/−^ Mice)

Liver-specific IGF-1-disrupted mice (LI-IGF-1^−/−^ mice) have very low serum IGF-1 levels and high serum GH levels due to inactivation of the IGF-1 gene. LI-IGF-1^−/−^ mice exhibited markedly decreased adiposity and as a result had 25% lower weight than wild-type mice. Only female LI-IGF-1^−/−^ mice showed a 16% increase in lifespan compared to that observed in control mice (34).

#### Pappa^−/−^ Mice

Pappa^−/−^ mice are the knockout for the pregnancy associated plasma A (PAPPA) gene, a specific protease for IGF binding proteins. The mean lifespan of this mouse strain was 38% longer compared to wild type controls. Pappa^−/−^ mice were dwarfs, but their serum glucose, insulin, IGF-1 and GH levels were not different from those of wild-type controls, suggesting that PAPPA acts mostly at autocrine or paracrine level and providing evidence for the role of local availability of IGF-1 in the modulation of longevity. In addition to extended longevity, Pappa^−/−^ mice showed a lower incidence of tumor development, as well as age related degenerative lesions (35, 36).

#### IRS Disrupted Mice

IRS-1 and -2 are important mediators for insulin, as well as for IGF-1 signaling. IRS1^−/−^ mice were insulin-resistant, with a defect in insulin signaling mainly in muscle tissue, about 30% smaller in size than the wild-type and only in females the lifespan was 18% longer compared with wild-type animals (37). IRS2^−/−^ mice were also insulin-resistant, but unlike IRS1^−/−^ mice, they exhibited defects in insulin signaling in more tissues, including the liver, the adipose tissues, and skeletal muscles. These mice developed diabetes, and had a much shorter lifespan than wild-type and IRS2^+/−^ mice. IRS2^+/−^ mice had improved insulin sensitivity and an increased lifespan (+18%) compared to wild-type mice. In addition, brain specific IRS2^+/−^ and IRS2^−/−^ mice were reported to be insulin resistant, and lived 18 and 14% longer than wild-type controls, respectively (38).

#### KLOTHO Modified Mice

Protein KLOTHO inhibits insulin and IGF-1 signaling, possibly by disrupting receptor/ligand interaction. Mice overexpressing KLOTHO were reported to have normal size, and males developed insulin resistance, while lifespan in both males and females was significantly increased (+18 and +30%, respectively) (39, 40).

#### P66shc Disrupted Mice (P66shc^−/−^ Mice)

P66shc is a protein mediating IGF-1 post-receptor signaling by activating the MAPK pathway. P66shc^−/−^ mice had normal phenotype, but lived 28% longer than wild-type controls (41). However, these data were not confirmed in a recent study (42).

The role of GH/IGF-1/insulin signaling in aging and longevity has been deeply studied through all these animal models. While in invertebrates the impact of downregulation in the IGF-1/insulin pathway on lifespan resulted to be clear and considerable, in murine models this effect was attenuated and not reproducible in some cases, such as in the IGF-1R^+/−^ and P66shc^−/−^ mice. However, most of these models showed the presence of some commonalities among the long-lived mice, such as reduced circulating IGF-1 and insulin levels and increased insulin sensitivity, which likely contribute to reduce tumor incidence, to improve stress resistance and to extend the lifespan. Genetic alterations able to disrupt IGF-1 system can keep the animals healthier for longer periods and can postpone or alleviate some age-related diseases. In this process nervous and adipose tissues seem to have a relevant role.

Additionally, more data are needed to determine the best time point during the lifetime for intervention in suppressing IGF-1 system to obtain beneficial effects on lifespan. In igf^f/f^ *C57B*l/6 mice deficiency in circulating IGF-1, starting at 5 months of age or earlier, increased lifespan by 15% only in females, with a reduction in the number of organs exhibiting disease pathology at the end of life compared to control group. Moreover, late-life IGF-1 deficiency (15 months) reduced cancer risk but had no beneficial effects on lifespan (43). These data underline the importance of IGF-1 deficiency when started early in life for increasing longevity. On the other hand, Mao et al. (44) recently reported that late treatment of 18-months old CB6F1 mice with an anti-IGF-1 receptor monoclonal antibody prolonged lifespan by 9% in females and improved several aspects of healthspan.

## IGF-1 System in Long-lived Individuals

Centenarians are considered the best human model to study biological determinants of longevity having reached the very extremes of the human lifespan (45).

Several studies compared circulating insulin and IGF-1 levels in centenarians with those of younger controls (46).

Metabolic age-dependent remodeling is a physiological process occurring in the whole population. Aging is frequently associated with a decline in glucose tolerance secondary to an increased insulin resistance (47), but an exception occurs in long-lived people. Paolisso et al. (48) found that insulin resistance increased with aging and declined in subjects older than 90 years living in Southern Italy. Indeed, long-lived subjects showed a higher insulin sensitivity and a better preservation of beta-cell function than younger subjects. Such difference was also independent of the main anthropometric and metabolic confounders. Centenarians had a lower 2-h plasma glucose concentration than that aged subjects (mean age 78 years) during oral glucose tolerance test. In centenarians insulin-mediated glucose uptake was greater than in aged controls during euglycemic glucose clamp, supporting a preserved glucose tolerance and insulin action in this long-lived group (49, 50). Similar results, supporting a better insulin sensitivity, were observed in other long-lived populations (51, 52).

Furthermore, centenarians showed a preserved insulin action not only on the glucose metabolism but also on adipose tissue. In fact, insulin infusion is normally associated with inhibition of lipolysis and thus to a significant decline in plasma free fatty acid and triglyceride concentrations. In centenarians the inhibitory activity of insulin on lipolysis was stronger than that of controls (mean age 78 years) (50). It is noteworthy that centenarians compared to aged controls have also a lower sympathetic tone which might be due to a better insulin action and thus, to a low fasting plasma insulin levels (53, 54).

Data on IGF-1 system in relation to longevity are still controversial in long-lived subjects (46). Paolisso et al. (55) described an increased plasma IGF-1/IGFBP-3 ratio in healthy centenarians compared to elderly subjects. They hypothesized that this elevated ratio was indicative of a higher IGF-1 bioavailability which contributed to the improved insulin action in centenarians. In contrast, Bonafè et al. (56) reported that subjects with at least an A allele of the IGF-1 receptor gene (G/A, codon 1013) had low levels of free plasma IGF-1 and were more represented among long-lived people. Arai et al. (57) described relatively low levels of serum IGF-1 in a population of Japanese centenarians. In this population the lowest tertiles of both IGF-1 and IGFBP-3 were associated with increased mortality (58).

These conflicting results probably reflect the complexity of the IGF-system and ethnic differences in enrolled populations. In addition, centenarians have often been compared to a control group of younger subjects. Therefore, in most of these studies it was not possible to conclude if IGF-1 differences between both groups were related to a different lifespan or reflected a physiological age-dependent IGF-1 decline. Indeed, there are several limitations to study centenarians: (1) low prevalence (1 centenarian per 5–10.000 inhabitants), (2) presence of frailty due to extreme age (almost 95% of centenarians have at least 1 frailty criterion), (3) lack of a control group of the same age (45, 59). Due to these limitations, this human model is unsuitable to study age-dependent variables that may be involved in the modulation of the lifespan.

Centenarians' offspring represent another interesting model to define relevant factors involved in human longevity and healthy aging. A concordant set of observations in different countries suggest that centenarian's offspring are healthier than members of the same demographic cohorts (51, 60, 61) and biologically (epigenetically) younger than their chronological age (62). Overall, these studies indicate that relatives of centenarians have a high probability for living longer and in good health (60, 63). In addition, studying centenarians' offspring has the relevant advantage of the availability of an appropriate demographically matched control group, consisting in age-matched offspring having both parents born in the same birth cohort of centenarians, but dead before the threshold age over which subjects were classified “long-lived.” This strategy is crucial for avoiding cohort effects. Therefore, centenarians' offspring model can overcome some limitations that are found in the study of centenarians (rarity, frailty and lack of an appropriate control) (60).

In few studies the IGF-1/insulin system has been characterized in centenarians' offspring and an appropriate matched control group.

We have evaluated circulating IGF-1 bioactivity, measured by an innovative IGF-1 Kinase Receptor Activation (KIRA) Assay in centenarians, centenarians' offspring and offspring matched-controls. Centenarians and centenarians' offspring had relatively lower circulating IGF-1 bioactivity compared to controls. Interestingly IGF-1 bioactivity in centenarians' offspring was inversely associated to insulin sensitivity (51).

Suh et al. (64) evaluated serum IGF-1 levels in Ashkenazi Jewish centenarians' offspring and in age-matched controls. Female centenarians' offspring had 35% higher serum IGF-1 levels than that controls. This difference may represent a compensatory response to reduced IGF-1 receptor signaling. Indeed, female offspring showed shorter stature than controls. In addition, an overrepresentation of heterozygous mutations in the IGF-1 receptor gene together with relatively high serum IGF-1 levels and weakened activity of the IGF-1 receptor has been described in Ashkenazi Jewish centenarians compared to controls without familial longevity.

In order to study longevity, other authors characterized these pathways in nonagenarian siblings and their offspring. In the Leiden Longevity Study, 421 families were recruited consisting of at least two long-lived Caucasian siblings, their offspring and partners of the offspring as control. In these populations serum glucose, insulin and triglycerides were the best biomarker of healthy aging (glucose and insulin low levels were considered healthy) (65). Nonagenarians in the lowest circulating IGF-1/IGFBP-3 ratio were associated with a better survival (66). The offspring of familial nonagenarians exhibited a better insulin sensitivity compared to their partner, while similar non-fasted serum levels of IGF-1 and IGFBP-3 were observed between both groups (67). Interestingly, 24-h total GH secretion was 28% lower in offspring compared with controls (68).

Another approach adopted to study longevity in humans consists in the selection of familial components of exceptional longevity and healthy aging, based on strict criteria, such as the Family Longevity Selection Score adopted in Long Life Family Study. These families enriched for exceptional life expectancy were compared to controls without family history of longevity (69). In this population circulating IGF-1 levels resulted to be a valid age-related biomarker (70).

In support of the potential role of the GH/IGF-1/insulin system in the human longevity, there are many genetic studies. Indeed, several genetic loci have been identified to be associated with circulating IGF-1 and IGFBP-3 levels and potentially able to affect aging (71). A genome-wide association analysis performed in nonagenarians and a population of subjects <60 years of age, showed a clear association between genetic variation of genes involved in insulin/IGF-1 pathway and human longevity (72). In a prospective study of older people, females with a genetic profile suggestive of a decreased insulin/IGF-1 signaling activity, exhibited a longer survival (73). In four independent cohorts of long-lived individuals it has been recently described a linear increased prevalence of GH receptor exon 3 deletion (d3-GHR) homozygosity with age. The presence of d3/d3 genotype increased life expectancy by about 10 years (74).

## IGF-1 System and Caloric Restriction

One of the most robust striking observations in the biology of aging is the capability of caloric restriction to prevent or delay several age-related diseases and to increase lifespan in mammals (75–78). The biological mechanisms of this phenomenon are not completely clear, but it has been suggested a potential involvement of relevant alterations in energy metabolism, endocrine system and oxidative damage.

Caloric restriction instigates numerous hormonal changes. In rodents caloric restriction without malnutrition suppressed circulating IGF-1 and insulin levels in proportion to the level of restriction, increased insulin sensitivity and resistance to stress and toxicity, and reduced the cancer risk (79, 80). Interestingly, most of these characteristics observed in wild type mice during caloric restriction resemble those reported in mice that are long-lived due to genetic disruption of the GH/IGF-1/insulin signaling, as previously described. In humans, randomized clinical trials showed that caloric restriction does not attenuate serum IGF-1 levels unless protein intake is reduced (81, 82). However, a recent meta-analysis, evaluating the effect of dietary restriction on well-recognized biomarkers of healthy aging, showed a decrease in circulating IGF-1 levels in humans (83). In addition, during caloric restriction skeletal muscle transcriptional profile showed a suppression of local insulin/IGF-1 pathway inducing a younger transcription profile (84).

Other circulating hormonal changes, such as decreased insulin, thyroid hormones and leptin levels, and increased adiponectin levels and insulin sensitivity have been observed during dietary restriction (85, 86). This hormonal adaptation may have a relevant role in extension of lifespan through several mechanisms:

1) *Reducing metabolic rate, cell proliferation, and oxidative stress*. In fact, IGF-1 is a potent growth factor and thyroid hormone is a potent stimulator of basal metabolic rate and oxidative metabolism. In addition, transcriptional patterns suggest that chronic moderate caloric restriction in adult individuals retards the aging process by shifting cellular metabolism from growth to maintenance and repair activities (84).
2) *Decreasing the accumulation of senescent cells*. Cellular senescence has been demonstrated to be a key mediator of aging (87). Over time protein homeostasis declines and damage accumulates. Interestingly, it is possible to delay several age-related diseases through attenuating the accumulation of senescent cells (88, 89). Normally the mTOR pathway is activated by several signals, including nutrients, IGF-1 and insulin (Figure 2). The down-regulation of this pathway, reported after caloric restriction, increased lifespan in several organisms. This effect seems to be secondary to an up-regulation of autophagy, a cytoprotective self-digestive process. In fact, autophagy is a cellular recycling process that can remove aged or damaged cellular components preventing the accumulation of senescent cells (90, 91).
3) *Counteracting inflammaging*. In both animals and humans dietary intervention can delay the aging process by attenuating low-grade inflammatory status (83, 92). The mechanisms underlying the anti-inflammatory activity of dietary restriction are not well-defined. It has been hypothesized that this effect is due to the reduction in fat mass and pro-inflammatory adipokines, and to an improvement of intestinal barrier integrity observed during dietary intervention (93, 94).

Interestingly, the endocrine biochemical profile observed in subjects during caloric restriction is comparable to that reported in centenarians, supporting a potential role of the endocrine system in the modulation of lifespan. In addition to an increase in insulin sensitivity and a decrease in plasma/serum IGF-1 levels, several studies showed an increase in circulating adiponectin levels and a reduction in circulating leptin and thyroid hormones levels in long-lived people compared to younger subjects (Table 1).

**Table 1:** Endocrine biochemical profile observed after caloric restriction and in centenarians compared to younger subjects.

**Endocrine parameters**	**Caloric restriction**	**Centenarians**
IGF-1	= /↓^*^	↓
Insulin	↓	↓
Insulin sensitivity	↑	↑
Adiponectin	↑	↑
Leptin	↓	↓
Triiodothyronine (T3)	↓	↓

↓, decrease; ↑, increase; =, no change;

* *more evident in murine models*.

Adipose tissue is an endocrine organ producing several cytokines involved in relevant processes, such as the energy metabolism, lipid, and glucose homeostasis and modulation of inflammatory response. Visceral adipose tissue has a main role in the development of metabolic diseases (95). Aging is associated with an increase in fat mass and a redistribution of adipose tissue, characterized by loss of peripheral subcutaneous fat and accumulation of visceral fat. In elderly, alterations in the secretion, synthesis and function of the adipokines have been described, probably due to an unbalance in the function, proliferation, size, and number of adipose cells (86). Adiponectin is an insulin sensitizing, anti-inflammatory and anti-atherogenic cytokine. Adiponectin circulates in the blood in several forms: trimer, hexamer, high molecular weight (HMW) multimer, and globular adiponectin (a proteolytically cleaved form). The HMW multimer is believed to be the more active form of adiponectin at protecting against insulin resistance and diabetes (96). Circulating adiponectin is independently and negatively related to facets of the metabolic syndrome, including insulin resistance, body weight, blood pressure, and serum lipids. Leptin is mainly produced in the subcutaneous and to a lesser extent in the visceral white adipose tissue. This cytokine regulates food intake, energy expenditure and atherogenesis. Leptin boosts weight loss by reducing appetite and stimulating metabolic rate and has pro-inflammatory properties (97).

Several studies reported that centenarians have higher plasma adiponectin and lower leptin concentrations than younger controls (53, 98–102). All forms of adiponectin were significantly increased in centenarians, but the HMW multimer was markedly higher (99). In centenarians the high adiponectin concentrations resulted to be independent of BMI, renal or cardiovascular function and were associated with a favorable metabolic phenotype (higher HDL-C, lower hemoglobin A1c, insulin, HOMA-IR and triglycerides) (98, 99). Increased adiponectin levels were also detected in the offspring of the long-lived subjects (older than 95 years) (103).

A decrease in thyroid hormones levels seems to be peculiar in centenarians. Mariotti et al. (104) reported that healthy centenarians had lower serum TSH and FT3 levels and higher serum rT3 levels compared with that observed in other control groups. In another Italian population of centenarians total T4 values were lower than normal range in 60% of examined subjects (105). Baranowska et al. reported that serum T3 levels in centenarians were lower compared with that observed in early elderly and young women (52). We have recently characterized thyroid function profile in an Italian cohort of 672 subjects (range 52–113 years old). An age-dependent decrease in FT3 level and FT3/FT4 ratio has been observed, while FT4 and TSH increased with aging (106). In Chinese centenarians' families a decline in thyroid function (high TSH and low FT3 concentrations) appears to be associated with age, and this phenotype is heritable (107). Corsonello et al. (108) found in relatives of centenarians (offspring or nieces/nephews) lower comorbidities, FT3, FT4, and TSH levels than age-matched controls who were not relatives of centenarians. In another Italian population lower plasma level of FT4 were observed in centenarians' offspring compared to age-matched controls (60).

In general, centenarians are lean (109) and follow healthy nutritional habits but without a calorie-restricted diet (110). Similarly to subjects during caloric restriction, a slower cell growing/metabolism, a better control in signal transmission and an enhanced autophagy have been observed in centenarians. Through a genome-wide DNA methylation analysis in centenarians and their offspring, we have identified epigenetically modulated genes and pathways potentially involved in the process of aging and longevity. Our results suggest that a better preservation of DNA methylation status, a slower cell growing/metabolism and a better control in signal transmission through epigenetic mechanisms characterized these populations (111). Centenarians have a preserved bioenergetic function through a mitochondrial hypertrophy that can recompense for functional defects (112). In addition, healthy centenarians have high levels of autophagy, as indicated by higher serum beclin-1 levels compared with both young patients with myocardial infarction and healthy controls (113). An increase in autophagic activity has been also observed in subjects belonging to families with exceptional longevity (114).

A relevant divergence occurs concerning the inflammatory status, which is attenuated in subjects after caloric restriction (115, 116) and high in centenarians (117–119). With aging a state of low-grade and chronic inflammatory condition (called inflammaging) and an increased prevalence of several diseases have been observed, such as cardiovascular disease, atherosclerosis, tumors, cognitive impairment, osteoarthritis, and diabetes (120, 121). Therefore, attenuation of chronic inflammatory status after caloric restriction represents a beneficial effect. Centenarians show signs of inflammaging but at the same time seem to be spared from its deleterious consequences. This apparent paradox can be explained by the fact that centenarians possess a complex and peculiar balancing between pro-inflammatory and anti-inflammatory factors, resulting in a slower, more limited and balanced development of inflammaging, in comparison with elderly, who are characterized by an inappropriate response to counteract chronic inflammation (120, 121).

These findings suggests common mechanisms to increase lifespan and to delay age-related diseases adopted in centenarians and in mammals following a calorie-restricted diet.

## Author's Opinion

Preclinical models have provided a great insight into the aging process with consistent data considering the role of the GH/IGF-1/insulin system in the modulation of lifespan. While it is well known that enhanced insulin sensitivity and low insulin levels are associated with an improved survival, there are several evidences showing that attenuation of the GH/IGF-1 axis may have beneficial effects in extending lifespan in humans. However, it is still unknown which are the optimal IGF-1 levels during life to live longer and healthier. In addition, IGF-1 receptor sensitivity and activation of the post-receptor pathway were not evaluated in the majority of the study enrolling long-lived subjects. Therefore, it is not possible to define the real activation status of the IGF-1 receptor signaling through the mere dosage of circulating IGF-1 levels. This renders more difficult the identification of pharmacological or environmental strategies targeting this system for extending lifespan and promoting healthy aging. A comprehensive understanding of these aspects remains a major challenge for uncovering interventions to slow human aging and to adopt in Rehabilitation Medicine. Future studies should evaluate the functional status of IGF-1 receptor signaling, also through transcriptional profiling and functional network analyses concerning IGF-1 regulated genes, in long-lived subjects.

## Conclusions

Striking similarities have been described concerning endocrine profile between centenarians and subjects after a calorie-restricted diet. The endocrine and metabolic adaptation observed in both models may be a physiological strategy to increase life span through a slower cell growing/metabolism, a slower loss of physiologic reserve capacity, a shift of cellular metabolism from cell proliferation to repair activities and a decrease in accumulation of senescent cells. These mechanisms seem to be, at least in part, mediated through the modulation of the GH/IGF-1/insulin system.

## Author Contributions

GP and MV researched all the data from available scientific literature on the PUBMED database. GV interpreted all data, organized, wrote, and revised the whole manuscript, and also conceptualized and drew all the figures assembling the final formatted review. LH organized and revised the whole manuscript.

### Conflict of Interest Statement

The authors declare that the research was conducted in the absence of any commercial or financial relationships that could be construed as a potential conflict of interest.
